# Supplementary material for: Precipitation induced by explosive volcanism on Mars and its implications for unexpected equatorial ice
Source: Nat Commun. 2025 Oct 14;16:8923. doi: 10.1038/s41467-025-63518-8 (PMC12521544; doi:10.1038/s41467-025-63518-8)
Supplement: Supplementary file 1 — Supplementary Information [file 41467_2025_63518_MOESM1_ESM.pdf]

Supplementary Materials

Supplementary Table 1: Summary of Sensitivity Tests

		Average rate of precipitation (kg m^{-2} s^{-1})		Maximum surface ice loading (kg m^{-2})	
Sensitivity Test	Input Parameter	Apollinaris Mons	Syrtis Major	Apollinaris Mons	Syrtis Major
Water mass eruption rate (MER)	10^6 kg s^{-1}	2.0×10^{-5}		5.2	
	10^7 kg s^{-1}	3.0×10^{-4}		74	
	10^8 kg s^{-1}	0.002		450	
	10^9 kg s^{-1}	0.005	0.001	1340	1430
Eruption duration	1 sol	0.005	0.001	460	490
	3 sols	0.005	0.001	1340	1430
	5 sols	0.005	0.001	2200	2350
Plume height	10^6 kg s^{-1} (MER)				
	35km	1.85×10^{-5}		4.9	
	45 km	1.95×10^{-5}		5.2	
	65 km	2.23×10^{-5}		5.9	
	10^7 kg s^{-1} (MER)				
	35km	2.7×10^{-4}		73	
	45 km	2.8×10^{-4}		74	
	65 km	1.5×10^{-4}		41	
	10^8 kg s^{-1} (MER)				
	35km	0.003		880	
	45 km	0.002		450	
	65 km	0.001		160	
	10^9 kg s^{-1} (MER)				
	35km	0.016	0.001	4200	4240
	45 km	0.005	0.001	1340	1430
	65 km	Model crash	Model crash	Model crash	Model crash
Obliquity	0°	0.001	0.001	1720	1660
	25.19°	0.001	0.001	1500	1590
	37.62°	0.005	0.001	1340	1430
	45°	3.0×10^{-4}	4.0×10^{-4}	1290	1390
	60°	3.0×10^{-4}	3.0×10^{-4}	1250	1330
H_2SO_4	35 km	2.7×10^{-4}		73	
	45 km	2.7×10^{-4}		73	
Season	Spring (L_S=0°)	0.005	0.001	1340	1430
	Summer (L_S=90°)	0.005		1340	
	Fall (L_S=180°)	0.005		1340	
	Winter (L_S=270°)	0.005		1340	
	Aphelion (L_S=71°)	0.005		1350	
	Perihelion (L_S=251°)	0.005		1340	
Ice albedo	0.5	0.005		1340	
	0.645	0.005	0.001	1340	1430
	0.95	0.005		1340	
CCN	10^4 kg s^{-1}	0.005		1310	
	10^5 kg s^{-1}	0.005	0.001	1340	1430
	10^6 kg s^{-1}	0.004		1180	
Topography	Pre-Tharsis bulge	1.1×10^{-4}	0.001	1280	1474
	Post-Tharsis bulge	0.005	0.001	1340	1430
1 year post-eruption	H_2SO_4: 35km	1.4×10^{-12}		72	
	No H_2SO_4: 35km	2.4×10^{-12}		60	
	H_2SO_4: 45km	1.9×10^{-12}		73	
	No H_2SO_4: 45km	2.9×10^{-12}		60	
	Ice albedo: 0.5	6.3×10^{-10}		1370	
	Ice albedo: 0.645	3.0×10^{-10}	6.2×10^{-11}	1360	1450
	Ice albedo: 0.95	1.0×10^{-10}		1350	
	Spring (L_S=0°)	3.0×10^{-10}	6.2×10^{-11}	1360	1450
	Summer (L_S=90°)	5.2×10^{-10}		1360	
	Fall (L_S=180°)	2.7×10^{-10}		1350	
	Winter (L_S=270°)	3.4×10^{-10}		1350	
	Aphelion (L_S=71°)	4.0×10^{-10}		1360	
	Perihelion (L_S=251°)	2.9×10^{-10}		1350	
	Obliquity (0°)	1.9×10^{-7}	1.1×10^{-7}	1780	1690
	Obliquity (25.19°)	9.6×10^{-8}	2.9×10^{-7}	1530	1620
	Obliquity (37.62°)	3.0×10^{-10}	6.2×10^{-11}	1360	1450
	Obliquity (45°)	7.1×10^{-8}	1.8×10^{-7}	1300	1410
	Obliquity (60°)	5.0×10^{-8}	1.2×10^{-7}	1250	1340
	Pre-Tharsis bulge	1.9×10^{-7}	7.4×10^{-11}	1289	1487
	Post-Tharsis bulge	3.0×10^{-10}	6.2×10^{-11}	1360	1450

The maximum surface ice loading and average rate of ice precipitation from sensitivity tests are presented. When each parameter is being individually varied, other parameters are set to baseline input parameters listed in Table 2. Blank entries mean no runs were performed. For sulfuric acid (H_2SO_4) sensitivity tests, the water and H_2SO_4 mass eruption rate are set to 10^7 and 10^5 kg s^{-1}, respectively. MER is the mass eruption rate and L_S is the solar longitude. The maximum surface ice loading for 1 year post-eruptions represent the maximum surface ice loading at the end of a 1 year simulation following the eruption. Precipitation rates and surface ice loadings are taken from the coordinates of the eruptive vent (174.4ºE, -8.5ºS for Apollinaris Mons and 67.17ºE, 9ºN for Syrtis Major).

Supplementary Table 2: Plume Heights						
Total mass eruption rate (kg s^{-1})	Water mass eruption rate (kg s^{-1})[a]	Total mass of water erupted (kg)	Plume heights used in this study	Hort and Weitz, (2001)[b]	Glaze and Baloga (2002)	Meyer et al., (2015)
10^8	10^6	8.9×10^{10}<br>2.7×10^{11}<br>4.4×10^{11}	35, 45, 65 km	32 km	37 km	37 km
10^9	10^7	8.9×10^{11}<br>2.7×10^{12}<br>4.4×10^{12}	35, 45, 65 km	–	~65 km	–
10^{10}	10^8	8.9×10^{12}<br>2.7×10^{13}<br>4.4×10^{13}	35, 45, 65 km	–	–	–
10^{11}	10^9	8.9×10^{13}<br>2.7×10^{14}<br>4.4×10^{14}	35, 45, 65 km	–	–	–

We test a range of plume heights to monitor its impact on the surface ice distribution and rate of precipitation. Plume height values used in this study for a given mass eruption rate correspond to the nearest PCM pressure level in a 1 bar atmosphere. The total mass of water erupted corresponds to the baseline duration of 1, 3, and 5 sols, respectively. [a]Value of the water mass eruption rate assuming a water content of 1 wt%[122]. [b]Value is for an initial volatile content of 1 wt%.

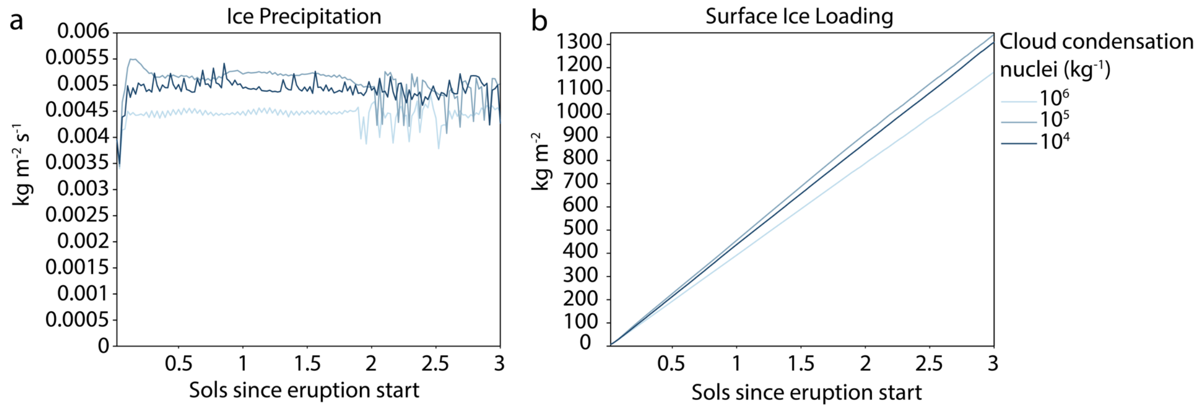

Supplementary Figure 1: Cloud condensation nuclei sensitivity test. Precipitation (a) and surface ice loading (b) are influenced by the amount of activated cloud condensation nuclei (CCN) per kg of air. Values for the amount of CCN correspond to amounts present for both water ice and vapor clouds. Values are taken from the eruptive vent of Apollinaris Mons located at 174.4ºE, -8.5ºS. Other parameters are set to baseline conditions as listed in Table 2.

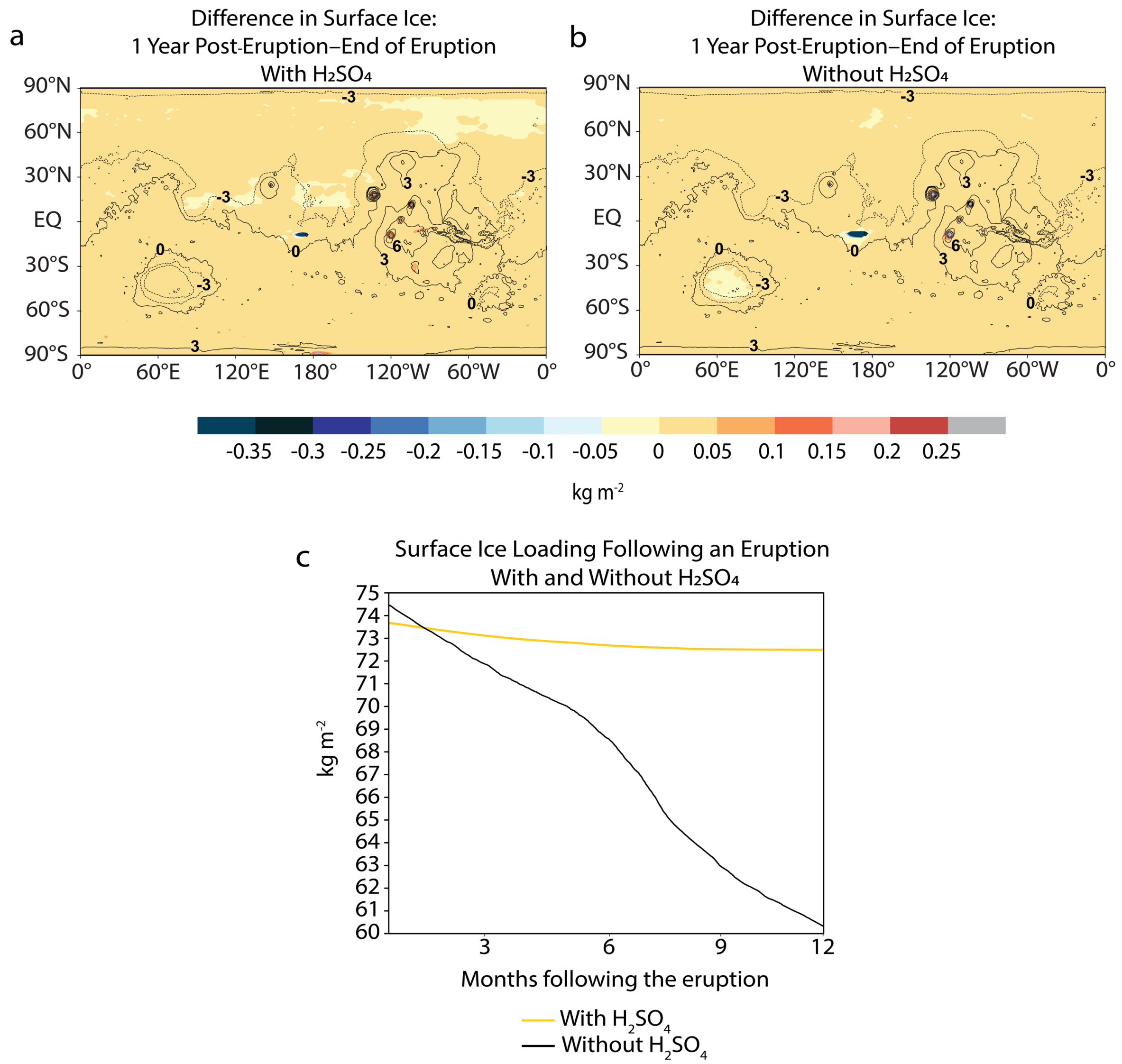

Supplementary Figure 2: Sulfuric acid (H_2SO_4) release from Apollinaris Mons protects surface ice against sublimation. (a–b) Values represent the difference in surface ice accumulation between the end of the eruption and one year post-eruption, with and without H_2SO_4. Negative values indicate net ice loss (sublimation) in a given region during the year following the eruption, while positive values indicate net ice accumulation. Eruption simulations are run under baseline conditions as listed in Table 2, except with a water mass eruption rate of 10^7 kg s^{-1}. In the case with H_2SO_4 present, the H_2SO_4 mass eruption rate is 10^5 kg s^{-1}. Modeled temperatures are overlaid onto a cylindrical projection of Mars. Black lines represent MOLA topographic contours, with numbers indicating contour elevations in kilometers. (c) The surface ice loading is taken from the coordinates of Apollinaris Mons located at 174.4ºE, -8.5ºS.
